# The Influence of the Lectin Pathway of Complement Activation on Infections of the Respiratory System

**DOI:** 10.3389/fimmu.2020.585243

**Published:** 2020-10-21

**Authors:** Anna S. Świerzko, Maciej Cedzyński

**Affiliations:** Laboratory of Immunobiology of Infections, Institute of Medical Biology, Polish Academy of Sciences, Łódź, Poland

**Keywords:** collectin, complement, ficolin, MASP, mannose-binding lectin, respiratory infection, MBL

## Abstract

Lung diseases are among the leading causes of morbidity and mortality. Complement activation may prevent a variety of respiratory infections, but on the other hand, could exacerbate tissue damage or contribute to adverse side effects. In this review, the associations of factors specific for complement activation *via* the lectin pathway (LP) with infections of the respiratory system, from birth to adulthood, are discussed. The most extensive data concern mannose-binding lectin (MBL) which together with other collectins (collectin-10, collectin-11) and the ficolins (ficolin-1, ficolin-2, ficolin-3) belong to pattern-recognition molecules (PRM) specific for the LP. Those PRM form complexes with MBL-associated serine proteases (MASP-1, MASP-2, MASP-3) and related non-enzymatic factors (MAp19, MAp44). Beside diseases affecting humanity for centuries like tuberculosis or neonatal pneumonia, some recently published data concerning COVID-19 are summarized.

## Introduction

Lung diseases are thought to be the 3^rd^ leading cause of morbidity and mortality worldwide. Recently, respiratory infections have been causing more deaths than previously expected due to the pandemic of coronavirus disease 2019 (COVID-19), the etiological agent of which is a betacoronavirus called severe acute respiratory syndrome coronavirus 2 (SARS-CoV-2). Complement activation may prevent or exacerbate lung injury. Therefore, investigation of the protective and harmful associations of complement factors in diseases of the respiratory system is crucial for understanding pathogenic mechanisms and establishing therapeutic strategies (1).

**Figure 1:**
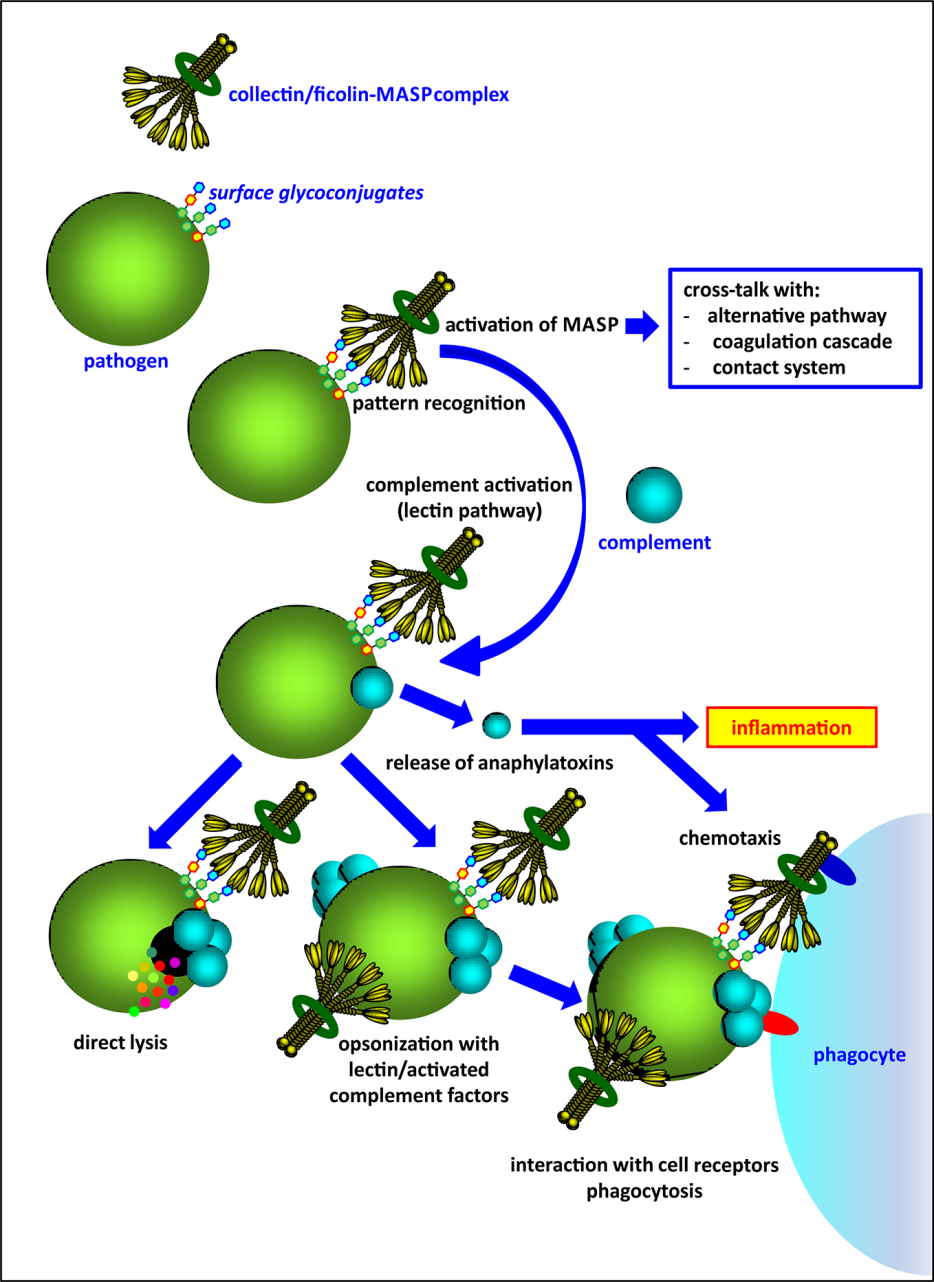
An overview of the activity of complement-activating collectins and ficolins, complexed with associated-serine proteases (MASP).

Activation of complement *via* the lectin pathway is initiated by several pattern-recognition molecules (PRM), complexed with mannose-binding lectin-associated serine proteases (MASP). These PRM are classified into two lectin families: collectins (mannose-binding lectin, MBL; collectin-10, CL-10 and collectin-11, CL-11) and ficolins (ficolin-1, ficolin-2, ficolin-3). As well as direct elimination of pathogens *via* complement-dependent lysis, they may act as opsonins and contribute to phagocytosis (**Figure 1**). Collectins and ficolins are structurally and functionally related. Their molecules are multimers of basic subunits, consisting of three polypeptide chains. Both collectins and ficolins are characterized by four domains: an N-terminal cysteine-rich region, a collagen-like region, an α-helical neck domain and a C-terminal functional domain. The last is a globular carbohydrate-recognition domain (CRD) (in collectins) or a fibrinogen-like (FBG) domain (in ficolins) responsible for ligand recognition and binding [reviewed in (2–4)]. Although it was initially believed that multimeric molecules of all complement-activating collectins and ficolins are built-up from identical polypeptides/subunits, it was demonstrated that collectin-10 (known also as collectin liver-1) and collectin-11 (or collectin kidney-1) may form heterocomplexes termed CL-LK (5–7). Later, Jarlhelt et al. (8) evidenced a similar phenomenon for ficolin-2 (L-ficolin) and ficolin-3 (H-ficolin or Hakata antigen). In both cases, heterocomplexes are suspected to have additional biological relevance (*i.e.* broader spectrum of ligands) compared with their parent molecules. MBL and CL-LK express a high affinity for D-mannose (D-Man), N-acetyl-D-mannosamine (D-ManNAc), N-acetyl-D-glucosamine (D-GlcNAc), D-fucose (D-Fuc), and L-fucose (L-Fuc) (2–4). Ficolins generally recognize acetylated compounds (not necessarily of carbohydrate nature), including N-acetyl-D-glucosamine, N-acetyl-D-galactosamine (D-GalNAc), and sialic acid but also D-galactose (D-Gal) (3). This wide repertoire enables interaction with numerous glycoconjugates on microbial surfaces like capsular polysaccharides, lipopolysaccharides, exopolysaccharides, fungal mannans or beta-glucans, viral glycoproteins, *etc*.

MBL recognizes a variety of respiratory pathogens, including *Staphylococcus aureus*, *Mycobacterium tuberculosis*, *M. avium* complex, *Haemophilus influenzae, Legionella pneumophila, Klebsiella pneumoniae, Mycoplasma pneumoniae, Chlamydia pneumoniae, Ch. psittaci, Ch. trachomatis, Nocardia farcinica, Aspergillus fumigatus*, influenza A virus and severe acute respiratory syndrome coronavirus (SARS-CoV-1). Furthermore, it binds to bacteria causing severe infections in cystic fibrosis (CF) patients like *Pseudomonas aeruginosa* and *Burkholderia cepacia* [reviewed in (9)] (**Table 1**). In the case of Streptococcus pneumoniae, efficient binding was originally observed only for non-capsulated strains since capsular polysaccharide abrogates recognition (10).

**Table 1:** Collectins and ficolins activating complement *via* the lectin pathway and their interactions with respiratory pathogens.

Family	Protein	Recognized respiratory pathogens
Collectins	Mannose-binding lectin (MBL)	*Staphylococcus aureus* (uncapsulated), *Mycobacterium tuberculosis*, *M. avium* complex, *Klebsiella pneumoniae*, *Haemophilus influenzae*, *Pseudomonas aeruginosa*, *Burkholderia cepacia*, *Legionella pneumophila*, *Mycoplasma pneumoniae*, *Chlamydia pneumoniae*, *Ch. psittaci*, *Ch. trachomatis*, *Nocardia farcinica*, *Aspergillus fumigatus*, influenza A virus, SARS-CoV-1
	Collectin-10 (CL-10)	Unknown; forms heterocomplexes with CL-11
	Collectin-11 (CL-11)	*M. tuberculosis*, *Str. pneumoniae* *K. pneumoniae*, *Ps. aeruginosa*, influenza A virus
Ficolins	Ficolin-1	*Staph. aureus*, *Str. pneumoniae*, *M. tuberculosis*, *Ps. aeruginosa*, *A. fumigatus*
	Ficolin-2	*Staph. aureus* (encapsulated), *Str. pneumoniae* (encapsulated), *H. influenzae*, *M. tuberculosis*, *Ps. aeruginosa*, *Pasteurella pneumotropica*, *A. fumigatus* (in cooperation with pentraxin-3) influenza A virus
	Ficolin-3	*M. tuberculosis*, *Pasteurella pneumotropica*, *A. fumigatus*, influenza A virus

References are given within the text.

Collectin-11 interacts with structures exposed on the surfaces of some Gram-positive and Gram-negative bacteria (including respiratory system pathogens: *M. tuberculosis, Str. pneumoniae, K. pneumoniae* and *Ps. aeruginosa*), fungi and influenza A virus (6, 11–14) (**Table 1**). It was also reported to recognize DNA and thought to participate in the response to apoptotic cells, neutrophil extracellular traps and biofilms (15). Moreover, CL-11 was believed to be involved in ischemic injury *via* recognition of L-fucose and subsequent complement activation at the site of ischemic stress (16, 17). Microbial targets recognized specifically by collectin-10 have not been reported to date; however, as a component of CL-LK, it is believed to extend its specificity and/or modify its affinity (4, 7). Both CL-10 and Cl-11 were demonstrated to contribute to the embryonic development, acting as chemoattractants for the cranial crest nerve cells. A variety of mutations of their genes (*COLEC10*, and *COLEC11*, respectively) are associated with the Malpuech, Michels, Mingarelli, and Carnevale (3MC) syndrome, manifested by craniofacial abnormalities [reviewed in (18)].

Ficolins are synthesized by variety of cells (ficolin-1 by neutrophils, monocytes and in bone marrow; ficolin-2 by hepatocytes; ficolin-3 by hepatocytes, alveolar type II pneumocytes and ciliated bronchial cells). They are present in the blood and contribute to the systemic immune response. Ficolin-1 (associated with lung macrophages) and ficolin-3 are furthermore involved locally in the respiratory system [reviewed in (3)].

Ficolin-1, known also as M-ficolin, binds to certain respiratory pathogens, as *Staph. aureus, Str. pneumoniae, M. tuberculosis, Ps. aeruginosa*, and *A. fumigatus* (**Table 1**). It recognizes some pneumococcal capsular polysaccharides [reviewed in (19)]. Ficolin-2 (L-ficolin) targets a relatively broad range of ligands (including bacterial lipoteichoic acids, capsular polysaccharides, fungal 1,3-*β*-glucans, DNA and elastin) *via* four binding sites in its FBG domain (20). Like ficolin-1, it is believed to participate in the clearance of late apoptotic cells (21, 22). *Staph. aureus, Str. pneumoniae* (in both cases encapsulated strains)*, H. influenzae, M. tuberculosis, Ps. aeruginosa, A. fumigatus*, and IAV are among the pathogens of the respiratory system recognized by ficolin-2. Furthermore, ficolin-2 binds to the surface structures of opportunistic *Pasteurella pneumotropica* [reviewed in (19)] (**Table 1**). Although the serum concentration of ficolin-3 (H-ficolin, Hakata antigen) was found to be the highest among PRM specific for the lectin pathway [its median level in healthy adults is 20 µg/ml or above (23–25)], few microbial targets have been reported. However, among those few are several respiratory pathogens: *A. fumigatus, M. tuberculosis*, IAV, and the afore-mentioned *Pasteurella pneumotropica* [reviewed in (19)] (**Table 1**). As mentioned, it was recently demonstrated that ficolin-3 and ficolin-2 may form heterocomplexes suspected to have more extensive biological relevance than the corresponding homooligomers (8).

These PRM are able to activate complement *via* the lectin pathway (LP) after complex formation with proteins of the MASP family, including three enzymes and two related proteins lacking proteolytic activity. As proenzymes, MASP exist as single polypeptide chains. Like C1r and C1s, which are involved in the activation of the classical pathway (CP), they include six domains (from N- to C-termini): CUB1 (C1r/C1s, urchin-epidermal, bone morphogenetic protein), EGF (epidermal growth factor), CUB2, CCP1 (complement control protein), CCP-2, and SP (serine protease, catalytic). When activated, the heavy (H) chain (CUB1–EGF–CUB2–CCP1–CCP2) and the light (L) chain (SP) are created (linked by a disulphide bond). The three N-terminal domains are responsible for MASP dimerization and complex formation with PRM [reviewed in (26)].

MASP-1, MASP-3, and the non-catalytic MBL-associated protein (MAp44 or MAP-1) are encoded by a single (*MASP1*) gene. MASP-1 undergoes auto-activation upon target recognition by collectins or ficolins. It is able to cleave C2 with low efficiency; therefore it was thought to upregulate LP activation. However, a key role of MASP-1 in MASP-2 activation was established (27). It moreover enables cross-talk with the coagulation and contact systems (28, 29), contributes to activation of platelets (30), endothelial cells, affects endothelial permeability (31–33), and regulates the transcription of complement factor D (34).

MASP-3 was first described to cleave insulin-like growth factor-binding protein-5 (IGFPB-5), regulating activity of insulin-like growth factors hence influencing cell proliferation, differentiation, motility, and survival (35). It was also thought to downregulate LP activation *via* competitive binding with MASP-2 to PRM. It was however evidenced that its major substrate is pro-factor D. Therefore, MASP-3 is directly involved in activation of complement *via* the alternative pathway (AP) (36–38). The MAp44 molecule comprises four domains in common with other products of the *MASP1* gene (CUB1–EGF–CUB2–CCP1) and 17 specific amino acid (AA) residues. Its biological role has not been established precisely; however, it is supposed to downregulate LP activation (*via* competition with MASP for formation of complexes with collectins or ficolins) [reviewed in (39)]. Furthermore, MAp44 was found to participate in regulation of cardiac development (40). The afore-mentioned CL-LK interacts with MASP-1 or MASP-3 homodimers (13, 41–43); therefore it may be supposed that neural crest cell migration depends on activity of those complexes. Indeed, a dozen *MASP1* mutations have been found associated with 3MC syndrome [reviewed in (18)].

MASP-2 protease and the non-catalytic MBL-associated protein MAp19 (known also as small MBL-associated protein, sMAP) are also products of a single (*MASP2*) gene. As the first mentioned cleaves C4 and C2, it is crucial for LP activation. Furthermore, it may contribute to the activation of the coagulation cascade, as its substrate is prothrombin [reviewed in (44, 45)]. MASP-2 cleaves kininogen as well; however, it is not associated with bradykinin release (29). Like MASP-1, it is involved in the regulation of transcription of factor D (34). MAp19 consists of CUB1 and EGF domains (shared with MASP-2) and four specific AA residues [reviewed in (44)]. It was thought to downregulate complement activation (*via* competition with MASP-2 for binding to pattern-recognizing molecules); however, that was not confirmed in a report published by Degn et al. (46).

As well as cross-talk between lectin and alternative pathways and with the coagulation and contact systems, ficolins and MBL were demonstrated to interact with long and/or short pentraxins contributing to the enhancement or regulation of the early immune response [reviewed in (47, 48)]. Previously, pentraxins were known to activate complement *via* the classical pathway, in association with C1q with no involvement of antibodies (48). The long pentraxin-3 (PTX3) was shown to co-operate with MBL, ficolin-1, and ficolin-2. On the other hand, it can interact with complement regulatory factors [C4-binding protein (C4bp), factor H] and therefore may contribute both to the amplification and modulation of complement activation [reviewed in (49, 50)]. Other (short) pentraxins recognize LP-associated PRM as well: C-reactive protein (CRP) was found to bind to ficolin-1 and -2, while serum amyloid protein (SAP) binds to MBL [reviewed in (47–49)].

Neutrophil extracellular traps (NET) are known to be important factors in host protection. Their release is promoted by complement-dependent opsonization of pathogens, including those invading the respiratory system [like *Str. pneumoniae, Staph. aureus, M. tuberculosis, A. fumigatus*, IAV, and respiratory syncytial virus (RSV)]. However, a variety of infectious agents, (for example pneumococci and *Staph. aureus*) developed mechanisms enabling them to escape from NET and promote dissemination from the upper to the lower respiratory tract and beyond (51, 52). Furthermore, when produced excessively, NET may contribute to harmful effects leading to airflow disturbance and chronic inflammation (51). NET-derived extracellular histones act cytotoxically and are mentioned among the main players of tissue damage. Their production may be induced by activation of complement and subsequent inflammatory processes (53). As lectin pathway-associated PRM are constitutively present in the respiratory system (ficolin-3 synthesized by type II pneumocytes and ciliated bronchial cell, ficolin-1 produced by lung macrophages) or are transferred from the bloodstream to the infected sites [MBL and ficolin-2 detected in bronchoalveolar lavage fluid (BALF) from patients suffering from pneumonia or invasive aspergillosis, respectively], they may contribute to excessive inflammation and its detrimental effects. Interestingly, PTX3 (a NET component) is considered to have a protective effect not only by contribution to trapping and killing pathogens but also by interacting with histones and other NET constituents (including myeloperoxidase and azurocidin 1), leading to modulation of the response (53, 54). Therefore, PTX3 appears a molecule enabling both boosting and mitigation of the early antimicrobial response, involved in its complement- and NET-related branches (53, 55). As mentioned above, it is able to amplify activation of the classical and lectin pathways (*via* interaction with their specific PRM) and downregulate each route (*via* binding of factor H and C4bp).

## Lectin Pathway-Associated Molecules in Respiratory Infections: From Newborn to Teenager

MBL was first believed to be protective against infections especially in infants and children aged 5–18 months (“window of vulnerability”) (56). However, numerous reports demonstrated associations of MBL deficiency with an increased susceptibility to various infections in newborns, older children/adolescents and adults (the last age group is discussed below).

Inherited lack of functional MBL is conferred by LXPA/O (referred to also as LXA/O or XA/O) and O/O genotypes. They correspond to six single nucleotide polymorphisms (SNPs) of the *MBL2* gene. Two of them are localized in the promoter region: −550 G>C (rs11003125, commonly called H/L) and −221 C>G (rs7096206, Y/X); one to the 5′-untranslated region (+4 C>T, rs7095891, P>Q). The coding region (exon 1) polymorphisms: +223 C>T (Arg52Cys, rs5030737), +230 G>A (Gly54Asp, rs1800450) and +239 G>A (Gly57Glu, rs1800451) are called A>D, A>B and A>C, respectively (D, B, and C variants are collectively designated O). Promoter SNPs affect the gene expression level (and thus MBL concentration in serum), while D/B/C alleles are related to markedly diminished ability to opsonize microbial cells and to activate complement. Those structural mutations lead to impaired oligomerization of subunits and diminished complex formation with associated serine proteases. Moreover, an increased sensitivity to endogenous metalloproteases is associated with shorter MBL half-life resulting in a lower serum level. As strong linkage disequilibria exist between the afore-mentioned polymorphisms, only seven haplotypes are commonly observed: HYPA, LYPA, LYQA, LXPA, HYPD, LYPB, and LYQC. Additionally, several rare variants (HXPA, LYPD, HYPB, LYQB) have been found in various populations [reviewed in (2)].

An adverse inﬂuence of MBL deﬁciency on risk of perinatal pneumonia, especially in premature babies was evidenced in several studies (57–60). A similar relationship was found for low ficolin-2 concentration in cord serum (58, 59). In contrast, babies with confirmed infections (mainly pneumonias) had higher ficolin-1 levels compared with newborns with no infections before leaving hospital (59).

Koch et al. (42) found that low MBL enhanced susceptibility to acute (mainly viral) infections of the respiratory system in infants aged 6–17 months supporting the “window of vulnerability” hypothesis. They suggested that in the younger (up to 5 months) population such an effect is moderated by maternal antibodies while in older (18–23 months) children, MBL dysfunction modifies disease course rather than influences infection risk itself (42). However, an important role of MBL in protection from pediatric respiratory infections of various etiologies was demonstrated also by Garred et al. (61) and Summerfield et al. (62). They found a relationship between O/O homozygosity and recurrent and/or severe pneumonias (61, 62). Our data indicated that insufficiency of MBL is associated with recurrence of infections of the respiratory system in children, especially when accompanied with other defects of the humoral response (63, 64). Furthermore, in two patients, the rare MASP-2 deficiency [related to +359 A>G (Asp120Gly or Asp105Gly in mature protein, rs72550870) homozygous mutation of the *MASP2* gene] was found. One of them suffered from recurrent pneumonias (63), while another had frequent infections of the upper respiratory tract and skin abscesses (64). The +359 A>G mutation, affecting the CUB1 domain, precludes formation of complexes with collectins and ficolins and therefore lectin pathway activation.

Low concentrations of ficolin-2 were also reported to enhance susceptibility to respiratory infections in children and teenagers (age range 1–16 years, mean 8.9), however, in the context of allergic rhinitis/asthma only. That association was not found in patients without allergic diseases. Based on those data, it was supposed that ficolin-2 may be protective against pathogens exacerbating allergic inflammation in the lung (64, 65). Ruskamp et al. (66) did not find any associations with nine *FCN2* gene SNP (located in promoter, introns, and coding region) or two intronic *FCN3* polymorphisms with respiratory system infections in a large cohort of children aged up to 4 years. It should be however, stressed that 6% of individuals only had frequent (>3/year) episodes. In our investigations (63–65), recurrence was defined as at least two pneumonias or serious sinus infections (requiring hospitalization) or at least eight upper respiratory tract infections within 12 months. Therefore the data published are not fully comparable.

Regarding the role of ficolin-1 in pediatric pneumonia, Elkoumi et al. (67) recently reported an association of (−144 C>A (rs10117466) SNP, localized in the promoter region of the *FCN1* gene. They found a higher frequency of the A/A genotype (and higher ficolin-1 serum levels) in children aged <5 years suffering from severe disease compared with controls and concluded that this protein may contribute to an enhanced inflammatory response resulting in a poorer outcome.

Bronchiectasis is an outcome of recurrent infections and may occur with or without cystic fibrosis (CF). The only study on children with non-CF bronchiectasis has been published recently (68). This study obtained negative results for *MBL2* alleles and haplotypes, while serum MBL concentrations were not determined.

## Lectin Pathway-Associated Molecules in Respiratory Infections: Adult

Roy et al. (69) reported *MBL2* O/O genotypes to be associated with a high risk of pneumonia and invasive pneumococcal disease in adults. However, this relationship was not confirmed by Kronborg et al. (70). Later, Gomi et al. (71) evidenced higher frequency of the *MBL2* B variant in Japanese patients suffering from recurrent respiratory infections compared with healthy controls. They furthermore evidenced the presence of mannose-binding lectin in bronchoalveolar lavage fluid from persons with active infection. Later, the presence of this lectin in BALF during infection was confirmed by Fidler et al. (72).

Collectin-11 and ficolin-2 (but not MBL) were shown to activate complement upon recognition of surface structures of *Streptococcus pneumoniae* (14). Consequently, Garcia-Laorden et al. (73) found no impact of MBL deficiency on the risk of development of community-acquired pneumonia (CAP) (commonly caused by pneumococci) or invasive pneumococcal disease. However, earlier the same authors postulated that MBL deficiency is associated with higher disease severity (including developing sepsis and multiorgan failure) and its fatal outcome (74), while Chalmers et al. (75) found no impact of low MBL serum concentration on CAP incidence or 1-month mortality. Ficolin-2 recognizes pneumococcal lipoteichoic acid, some capsular polysaccharides of pathogenic serotypes and pneumolysin (the major toxin of those bacteria) (10, 76–78). It was moreover suspected that the low invasiveness of *Str. pneumoniae* 11A serotype is related to recognition of its capsular polysaccharide by ficolin-2 (79). Nevertheless, Chapman et al. (80) found no relationship between *FCN2* gene polymorphisms and invasive pneumococcal disease. Chalmers et al. (75) noticed an association of very low ficolin-2 serum levels (<1,200 ng/ml) with higher risk of 30-day mortality in CAP patients. The data discussed above suggest that ficolin-2 may be protective against infections with some *Str. pneumoniae* strains, depending on a variety of factors, including bacterial serotype and the *FCN2* genotype/ficolin-2 serum concentration in the host. Moreover, MASP-2 primary deficiency was reported to be associated with severe pneumococcal pneumonia and to modify the course of cystic fibrosis (43, 81, 82).

Interestingly, van Kempen et al. (83) reported that *MBL2* genotypes conferring high gene expression levels (YA/YA, YA/XA) predispose to CAP caused by intracellular pathogens (*Coxiella burnetii, Legionella* spp., *Chlamydia* spp. *Mycoplasma pneumoniae*), supposedly by the contribution of MBL to enhanced phagocytosis. However, low MBL-dependent complement activity was earlier shown to be a risk factor for legionnaires’ disease (84). Furthermore, +6424 G>T (rs7851696) *FCN2* gene polymorphism minor allele (related to low ficolin-2 serum concentration) was observed to be a risk factor for *C. burnetii* pneumonia (83).

A few cases of the rare ficolin-3 deficiency associated with +1637 C>delC (rs28357092) variant allele homozygosity have been reported. In one of them, it was found associated with numerous severe/recurrent infections, including those affecting the respiratory system. The adult patient suffered from recurrent lower respiratory tract diseases from early childhood. As a young adult, he was hospitalized due to bilateral frontal cerebral abscesses caused by non-hemolytic streptococci and several times due to pneumonia (*H. influenzae* and *Ps. aeruginosa* were identified as aetiological agents). Furthermore, severe bronchiectasis, pulmonary fibrosis, and obstructive lung disease were diagnosed (85).

Haerynck et al. (86) found an association of the A variant [related to *MASP1* gene +1851 G>A (rs3821805) SNP, localized in exon 12, encoding for MASP-3 SP domain) with earlier onset of chronic *Ps. aeruginosa* colonization in cystic fibrosis patients. Interestingly, the presence of the A allele does not affect the protein sequence (Leu617Leu). It was therefore supposed it may influence mRNA splicing, its stability, structure, and protein folding (86).

Numerous studies have investigated the role of lectin pathway factors in susceptibility to pulmonary tuberculosis. The majority of them concerned MBL. As revealed in several meta-analyses, the role of MBL in that disease is unclear and inconsistent, apparently reflecting differences in study design, ethnicity and number of patients tested. Although it is generally believed that *MBL2* O alleles may contribute to enhanced susceptibility to TB, at least in some populations (87–89), certain data suggest diverse associations for D, B, and C variants. Tong et al. (90) concluded that minor alleles in codons 52 (D) and 54 (B) elevate the risk of developing disease while that in codon 57 (C) has a protective effect (in Chinese population). In contrast, Mandal et al. (91) postulated an unfavorable effect of the C allele and a beneficial effect of the D allele. The protective role of ficolin-2 from tuberculosis (caused by *M. tuberculosis*) and pulmonary infection with *M. avium* complex was reported by Luo et al. (92) and Kobayashi et al. (93), respectively.

Recently, we reported a significantly higher frequency of *MBL2* O variants among adults diagnosed with pulmonary tuberculosis (PTB), compared with controls. Furthermore, investigation of the *FCN1* gene −542 G>A polymorphism revealed a higher incidence of G/G homozygosity among patients. The median concentration of MBL in serum was significantly higher in the disease group, despite afore-mentioned genetic association (94). Similarly, median ficolin-1 level was higher in patients (94), although the *FCN1* G allele at position −542 was earlier reported to be associated with the opposite effect (95). In contrast, ficolin-3 concentrations were generally lower among persons with confirmed infection. Furthermore, a high potential of ficolin-1 to differentiate between PTB patients and controls was noted; therefore, it was suggested to be considered a supplementary marker of active tuberculosis. Furthermore, two patients were found to be MASP-2-deficient (94).

The *MBL2* D allele was suggested to contribute to higher susceptibility to pulmonary chronic necrotizing aspergillosis in British adults (96). Furthermore, Bidula et al. (97) observed significantly higher BALF ficolin-3 concentrations in patients with confirmed pulmonary *A. fumigatus* infection compared with controls and considered this lectin to be a promising disease marker. Interestingly, both ficolin-2 and pentraxin-3 recognize *A. fumigatus* independently; however they were shown to recruit each other to the pathogen surface in a calcium-dependent and synergistic manner (98). That leads to the cross-talk between classical and lectin pathways and amplification of ficolin-2-dependent complement activation (98). As ficolin-2 was detected in BALF from patients suffering from invasive aspergillosis (99), it was supposed that it may enhance complement-mediated phagocytosis (48). Furthermore, PTX3 deficiency in donors was found associated with enhanced risk of that disease in recipients of allogeneic hematopoietic stem cell transplants (100) supporting the conclusion that the cross-talk between PTX3 and ficolin-2 may boost anti-fungal response in the lung (50).

Due to interaction with hemagglutinin and neuraminidase, MBL is able to neutralize influenza A virus (IAV) in a complement-independent way (101–103). However, no role for MBL in protection from the disease has been reported to date. Regarding other respiratory viral infections, low MBL concentration in serum was reported to be associated with higher risk and more severe course of respiratory syncytial virus (RSV) disease in children (104). Furthermore, the *MBL2* B variant was associated with the severe acute respiratory syndrome (SARS) (105, 106). On the other hand, Yuan et al. (107) found no relationship with any *MBL2* SNP in SARS.

There is also some evidence that MBL insufficiency (defined genetically or at the protein level) affects the course and severity of CF-associated bronchiectasis, although not manifested clinically in childhood (108). Similar results were obtained with non-CF bronchiectasis adult patients (109) although not confirmed by another group (110).

## Possible Associations of Complement Activation *via* the Lectin Pathway With COVID-19

The current pandemic of coronavirus disease 2019 (COVID-19) is caused by severe acute respiratory syndrome coronavirus 2 (SARS-CoV-2). Non-glycosylated SARS-CoV-2 nucleocapsid protein (like corresponding components of SARS-CoV-1 and MERS-CoV) was demonstrated to activate complement *via* the lectin pathway (111). Data reported by Magro et al. (112) evidenced depositions of MASP-2, co-localized with C4d and SARS-CoV-2 spike glycoprotein (S-gp) in septal capillaries and interalveolar septa of the lungs from COVID-19 non-survivors. Recently, recombinant MBL has been mooted as a therapeutic agent, acting *via* inhibition of binding of S-gp to the angiotensin converting enzyme 2 (ACE2) cell receptor and by promoting phagocytosis (113).

The RNA sequence of the better-studied SARS-CoV-1 is highly homologous to that of SARS-CoV-2, and both viruses recognize the same human receptor. Ip et al. (105) reported that MBL bound to SARS-CoV-1 and enhanced C4 deposition on the viral surface. They also found that this lectin is able to inhibit the infectivity of SARS-CoV-1 in fetal rhesus monkey kidney cells. In addition, MBL seems to recognize SARS-CoV-1 S-gp which results in preventing infection (114). However, complement activation may also be detrimental. Based on data from a murine model, Gralinski et al. (115) suggested complement activation contributed to acute respiratory distress syndrome (ARDS) associated with SARS-CoV-1 infection: C3^−/−^ mice had reduced lung neutrophilia and less severe systemic inflammatory response.

Similar findings are emerging for SARS-CoV-2 and COVID-19. Complement activation may provoke a “cytokine storm” leading to ARDS and organ failure. That supposition was first supported by promising data from patients treated with complement inhibitors (116–119). Polycarpou et al. (120) suggested that targeting complement might contribute to reducing COVID-19 systemic complications (multiorgan failure, coagulopathy), mediated by lectin pathway activation. Therefore, inhibition of the complement system at the levels of C3 or C5a/C5aR is considered a possible therapeutic option (121, 122).

Matricardi et al. (123) proposed an intriguing model explaining the course of SARS-CoV-2 infection. They suggested an efficient local innate immune response (MBL, natural antibodies *etc*.) may eliminate the pathogen. However, when the virus replicates and spreads, it induces a strong adaptive response (with involvement of specific IgM and IgG) leading to severe inflammation with involvement of complement and coagulation cascades, resulting in a “cytokine storm” (123). Thus, complement activation seems to be beneficial at the early stage of infection but it may be severely harmful at a later stage. It should be stressed that MBL-dependent complement activation may be associated with adverse effects as well, leading to the amplification of an excessive response. Eriksson et al. (124) reported that MBL contributed to pathological thrombosis and coagulopathy (but not other organ dysfunction or intensity of inflammation) in critically ill COVID-19 patients. In contrast, Holter et al. (125) did not find much difference in MBL concentrations in plasma between COVID-19 patients and controls although a transient increase of its level (at days 3**–**5 after hospital admission) was noted. It should be however stressed that narsoplimab, specifically targeting MASP-2, has recently been demonstrated to be a promising therapeutic agent, reducing detrimental effects of complement activation and giving no adverse reactions itself (126).

## Concluding Remarks

The findings reviewed here detail a variety of associations between factors specific for complement activation *via* the lectin pathway and infections of the respiratory system, from birth to adulthood. However, they are not entirely consistent (especially those concerning pulmonary tuberculosis) and therefore have to be considered in relation to ethnic, geographical and social backgrounds, study design, number of patients recruited, *etc*. Nowadays, extensive investigations concerning the lectin pathway (and complement in general) associations with pandemic SARS-CoV-2 infection have to be considered crucial as they may result in elaboration of efficient treatment strategies.

## Author Contributions

MC and AŚ conceptualized the background of this review, collected, and selected literature to be discussed. MC wrote draft manuscript. AŚ reviewed the draft version. MC prepared the submitted version. All authors contributed to the article and approved the submitted version.

## Funding

This work was partially supported by National Science Centre, Poland, grant UMO-2015/17/B/NZ6/04250 and Institute of Medical Biology, Polish Academy of Sciences.

## Conflict of Interest

The authors declare that the research was conducted in the absence of any commercial or financial relationships that could be construed as a potential conflict of interest.
